# The First Comprehensive Evaluation of Immuno-Inflammatory Markers for Prognosis in Esophageal Cancer Patients: A South Asian Perspective

**DOI:** 10.3390/clinpract14050163

**Published:** 2024-10-06

**Authors:** Sajida Qureshi, Waqas Ahmad Abbasi, Hira Abdul Jalil, Saba Mughal, Muhammad Saeed Quraishy

**Affiliations:** 1 Dow Medical College, Dow University of Health Sciences, Karachi 74200, Pakistan; waqas.abbasi@duhs.edu.pk (W.A.A.); dr.hkhan9@gmail.com (H.A.J.); saeedqur@gmail.com (M.S.Q.); 2 School of Public Health, Dow University of Health Sciences, Ojha Campus, Karachi 75300, Pakistan; saba.mughal@duhs.edu.pk

**Keywords:** esophageal cancer, prognosis, inflammation, South Asia

## Abstract

**Background:** Esophageal cancer (EC) remains a significant health challenge in South Asia, with poor prognosis despite advancements in diagnostics and treatment. Identifying and validating prognostic factors is essential for improving patient outcomes. **Methods:** A prospective study was conducted with 146 biopsy-confirmed EC patients at the Dr. Ruth K.M. Pfau Civil Hospital, Karachi, Pakistan. Clinical and laboratory data were collected and analyzed using descriptive statistics, receiver operating characteristic (ROC) analysis, and the Chi-square test. Survival outcomes were assessed using Kaplan–Meier curves, log-rank tests, and Cox proportional hazard models for univariate and multivariate regression analyses, with statistical significance set at *p* ≤ 0.05. **Results:** Bivariate analysis showed significant associations of the neutrophil lymphocyte ratio (NLR) (*p* = 0.017), C-reactive protein to albumin ratio (CAR) (*p* = 0.033), red cell distribution width to platelet ratio (RPR) (*p* = 0.020), and systemic immune-Inflammation index (SII) (*p* = 0.009) with patient survival. Univariate analysis identified tumor length >10 cm (*p* = 0.016), T4 stage (*p* = 0.015), metastasis (*p* < 0.001), surgery not performed (*p* < 0.001), and SII (*p* = 0.022) as significant factors for survival, with higher SII linked to poorer overall survival (*p* = 0.020). Interestingly, in the multivariate model, only metastasis (*p* < 0.001) and surgery not performed (*p* = 0.011) remained significant. **Conclusions:** Immuno-inflammatory markers may be less pertinent prognostic factors for EC in the South Asian population.

## 1. Introduction

The incidence of esophageal cancer (EC) has increased worldwide in recent decades, leading to significant morbidity and mortality, ranking as the sixth leading cause of cancer-related deaths globally. Moreover, Asia has the highest incidence and mortality rates for EC among all continents. In Pakistan, it is the fourth most prevalent cancer and is rising exponentially [1,2]. Despite advancements in diagnostic techniques and treatment modalities over the past two decades, the prognosis for EC remains poor, with a five-year survival rate below 20%. This poor prognosis is attributed to several factors, including delayed diagnosis, low public awareness, and late referrals to specialized healthcare centers [3,4]. Therefore, it is crucial to identify factors that influence disease progression, tumor characteristics, and overall prognosis.

Systemic inflammation plays a key role in cancer development and progression, while tumor-associated inflammation contributes to poor patient outcomes [5]. Numerous studies have demonstrated the prognostic significance of multiple immuno-inflammatory indices in various cancers [6,7,8,9]. These indices are a cost-effective means to evaluate the tumor progression state and are easily incorporated into routine clinical assessments. Recent studies have specifically evaluated the prognostic usefulness of the neutrophil lymphocyte ratio (NLR), platelet lymphocyte ratio (PLR), c-reactive protein to albumin ratio (CAR), lymphocyte monocyte ratio (LMR), modified Glasgow prognostic score (mGPS), red cell distribution width to platelet ratio (RPR), systemic immune-inflammation index (SII), and systemic inflammation score (SIS) in patients with EC [10,11,12,13,14,15,16]. However, a comprehensive prospective evaluation of these inflammatory indices collectively is lacking, particularly in the context of the South Asian population [12]. Therefore, this study aims to thoroughly identify the prognostic implications of these markers in EC patients, aiming to validate their combined significance in predicting survival outcomes and thereby guiding management decisions.

## 2. Materials and Methods

### 2.1. Study Design and Setting

All subjects provided informed consent. The study was conducted according to the guidelines of the Declaration of Helsinki, and approved by the Institutional Review Board of DUHS (IRB-2672/DUHS/26/10/2022), and employed a prospective study design. Patient recruitment took place over one year, with participants being followed for an additional year from the date of enrollment. The study was conducted at the Department of Upper GI Surgery, Surgery Unit-I, Dr. Ruth K.M. Pfau Civil Hospital in Karachi, a prominent government-sector tertiary care facility.

### 2.2. Sample Size, Inclusion, and Exclusion Criteria

The sample size was calculated using the OpenEpi calculator, with a 95% confidence interval and a 5% margin of error based on an estimated population size of 70 patients over a two-year period. This estimation was informed by prior patient intake records. Using the prevalence of NLR ≥ 2.38 in EC reported at 50.8% [17], the calculation yielded a minimum required sample size of 60 patients. Inclusion criteria included patients with biopsy-proven EC, including both squamous cell carcinoma and adenocarcinoma, that have not undergone neoadjuvant treatment. Exclusion criteria encompassed patients with a history of chronic inflammatory or autoimmune disorders or recent infections that could influence inflammatory markers, as well as prior malignancies, including recurrent EC along with other concomitant malignancies, and those who left against medical advice. During the study period, patient recruitment proceeded at a faster rate than anticipated. Consequently, to maximize the robustness of our analysis, all 146 patients who met the inclusion criteria and were admitted during the study enrollment period were included in the final analysis.

### 2.3. Data Collection

Informed written consent was obtained from each patient prior to data collection. Patient-specific details, including gender, age, tumor location, tumor length, histological type, depth of tumor (T), the involvement of nodes (N), distant metastasis (M), and overall clinical stage (cTNM), were collected through reviews of biopsy, endoscopy, CT scan, and PET scan results. Laboratory data were uploaded and sourced from the Hospital Information and Management Security System (HIMSS).

Inflammatory markers, including NLR, PLR, LMR, CAR, and RPR, were calculated based on complete blood count results. The mGPS was derived using serum albumin and CRP levels. Patients with elevated CRP (>10 mg/L) and hypoalbuminemia (<35 g/L) received a score of 2, those with hypoalbuminemia (<35 g/L) and CRP (≤10 mg/L) received a score of 0, and those with elevated CRP (>10 mg/L) and albumin (≥35 g/L) received a score of 1 [18].

The SIS was derived using serum albumin levels and LMR. Patients with both increased LMR (≥4.44) and increased albumin (≥35 g/L) received 0 as a score. Those with either increased LMR or increased serum albumin were given a score of 1. Patients with decreased albumin (<35 g/L) and decreased LMR (<4.44) were assigned a score of 2 [13]. The SII was calculated using the following formula: platelet count multiplied by the ratio of neutrophil count divided by lymphocyte count [19].

Furthermore, all patients received standard treatment plans, including chemoradiotherapy. Among all participants, those with resectable cancers underwent minimally invasive esophagectomy (MIE). Patients who were not candidates for surgery, either due to unresponsiveness to chemoradiotherapy or uncontrolled metastasis, were referred for palliative care. Follow-ups were conducted monthly for one year, with OS as the primary outcome variable of interest. Laboratory assessments were conducted at a single diagnostic facility, ensuring consistent and standardized evaluation of markers.

### 2.4. Statistical Analysis

Descriptive statistics, such as frequency, percentage, median, and interquartile range, were reported. A receiver operating characteristic (ROC) examination was used to calculate the area under the curve (AUC) and to identify the optimal cut-off values of inflammatory markers for predicting OS. Bivariate associations of clinicopathological characteristics of patients were examined with patients’ status (alive or expired) using the Chi-square test. OS was measured from the date of cancer diagnosis to the last follow-up, which occurred one year after enrollment. Kaplan–Meier survival curves were generated, and differences between groups were assessed using the log-rank test. Univariate and multivariate regression analyses were taken into account with a Cox proportional hazards model and hazard ratios (HRs) with 95% confidence intervals. A *p*-value of ≤0.05 was considered statistically significant. Data analysis was performed using SPSS version 24.

## 3. Results

### 3.1. Patient Characteristics

The study included 146 patients with a median age of 45 years, ranging from 20 to 80 years. The optimal cut-off values for the markers predicting OS were determined as follows: 3.6 for NLR, 198.4 for PLR, 4.3 for LMR, 0.17 for CAR, 5.2 for RPR, 858 for SII, and 2 for both SIS and mGPS (Table 1). Among these patients, 53% (*n* = 77) were females and 47% (*n* = 69) were males. The majority of the patients have squamous cell carcinoma at 73.3% (*n* = 107) compared to adenocarcinoma 26.7% (*n* = 39). The most common location of the tumor was in the lower thoracic esophagus at 54.1% (*n* = 79), followed by the middle thoracic 37% (*n* = 54) and upper thoracic esophagus at 8.9% (*n* = 13). Most of the cases (65.1%) (*n* = 95) were moderately differentiated, followed by 24.7% (*n* = 36) who were poorly differentiated, and 10.3% (*n* = 15) who were well differentiated. The distribution of tumor length groups was given as 30.1% (*n* = 44) in the <5 cm group, 53.4% (*n* = 78) in the 5 to 10 cm group, and 16.4% (*n* = 24) in the >10 cm group.

Based on the cT stage, 13.0% (*n* = 19), 43.2% (*n* = 63), and 43.8% (*n* = 64) of cases had T1&T2, T3, and T4 stages, respectively. The distribution of lymph node involvement was as follows: the proportion of the regional lymph node involved (N0) was 8.9% (*n* = 13), followed by N1 in 34.9% (*n* = 51), N2 in 30.1% (*n* = 44), and N3 in 26% (*n* = 38) of the patients. Metastasis was present in 70.5% (*n* = 103) of cases. The proportion of cTNM distribution was 8.2% (*n* = 12) for stages I and II, 31.5% (*n* = 46) for stage III, and 60.3% (*n* = 88) for stage IV (Table 2).

### 3.2. Patient Outcomes and Associations

Eighty-seven (60%) deaths were observed and it was noted that patients who were expired were more likely to have a higher tumor length (23% vs. 7%, *p*-value = 0.031), higher T-stage (49% vs. 35%, *p*-value = 0.021), N stage (34% vs. 13%, *p*-value < 0.001), M stage (42% vs. 10%, *p*-value < 0.001) and clinical stage (70% vs. 46%, *p*-value = 0.012) compared to those who were alive. Likewise, surgery status, when not conducted (82% vs. 58%, *p*-value = 0.002), higher values of NLR ≥ 3.6 (54% vs. 34%, *p*-value = 0.017), CAR ≥ 0.17 (82% vs. 66%, *p*-value = 0.033), RPR ≥ 5.2 (76% vs. 57%, *p*-value = 0.020), and the SII index ≥ 858 (69% vs. 47%, *p*-value = 0.009), were also positively associated with the death status of patients. However, PLR, LMR, SIS, and mGPS did not show any significant findings (Table 2).

### 3.3. Survival Analysis and Prognostic Factors for Overall Survival in Esophageal Cancer Patients

**Figure 1:**
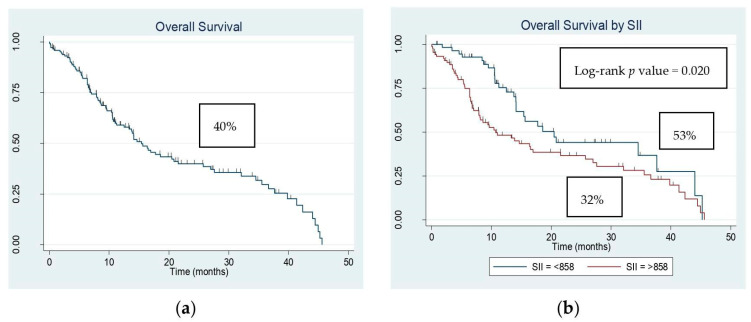
Kaplan–Meier estimates of overall survival. (**a**) Kaplan–Meier estimate of overall survival for all patients. (**b**) Kaplan–Meier estimate of overall survival based on the systemic immune-inflammation index (SII).

Median follow-up time was 10.5 months, which ranged between 0.03 and 45.6 months. The median OS was 11.0 months, and the OS rate of patients was 40% (*n* = 59) (Figure 1a). It was noted that higher SII (OS: 32% vs. 53%, log-rank *p*-value = 0.020) was associated with the poor OS of patients (Figure 1b). Univariate Cox regression model revealed that patients a with higher M stage (HR = 4.3, 95% CI: 2.7–7.0, *p*-value < 0.001), with surgery not performed (HR = 2.86, 95% CI: 1.65–4.95, *p*-value < 0.001) and SII ≥ 858 (HR = 1.7, 95% CI: 1.1–2.7, *p*-value = 0.022) were significantly associated with an increased risk of mortality compared to those who had a lower M stage, surgery conducted, and SII < 858. The multivariate model was adjusted for those covariates who had a *p*-value < 0.25 in univariate analysis. No significant association of inflammatory markers could be obtained in the multivariate analysis for OS, but patients with a higher M stage (HR = 3.1, 95% CI: 1.6–5.7, *p*-value < 0.001) and those who did not undergo any surgery (HR = 2.3, 95% CI: 1.2–4.2, *p*-value = 0.011) remained at high risk of mortality (Table 3).

## 4. Discussion

EC is a major global health issue, and predicting prognosis using pre-operative factors is crucial for determining peri-operative treatment strategies [12]. The systemic inflammatory response, linked to the inhibition of apoptosis, angiogenesis, and DNA damage, contributes to tumor progression and metastasis [9]. Recent studies have increasingly acknowledged the role of systemic inflammatory responses in influencing both short- and long-term outcomes across various cancers [20]. To our knowledge, this is the first prospective study in South Asia, particularly Pakistan, that thoroughly examines the prognostic value of immune-inflammatory markers, such as NLR, PLR, CAR, LMR, mGPS, SII, SIS, and RPR. Our findings highlighted the significance of several markers, particularly the prominence of higher SII levels (≥858). However, it is noteworthy that no significant association of these markers was observed in the multivariate analysis for OS, which presents an interesting contrast specific to the South Asian population.

In a meta-analysis by Jiang et al. [21], which included 72 studies, pretreatment levels of CAR and mGPS were shown to have an outstanding predictive value for 5-year OS in EC. Similarly, Chen et al. [22] identified that a low LMR (<4) is an actual predictor of poor EC survival. Binfeng et al. [23], in a meta-analysis of 32 studies involving 8431 patients, concluded that elevated NLR values are associated with poor prognosis in EC. Additionally, SIS, based on pretreatment serum albumin and LMR, as well as PLR, have been identified as independent prognostic factors in EC [24,25]. Among these markers, the mutual prognostic value of RPR remains poorly established in cancers, including EC. Hu et al. [26] highlighted RPR as superior to other blood-routine markers, demonstrating a strong prognostic capability for mortality in esophageal squamous cell carcinoma (ESCC). In our analysis, NLR (*p* = 0.017), CAR (*p* = 0.033), and RPR (*p* = 0.020) were significantly related to patient status (alive or dead) (Table 2).

Moreover, the prognostic value of SII has been widely recognized in recent studies. Zhang et al. [19] demonstrated that a high preoperative SII (≥387.65) is a strong indicator of aggressive tumor biology and poor prognosis. While most of these studies have focused on ESCC, our exploration, which included both ESCC and adenocarcinoma, revealed that patients with a higher SII (≥858) had significantly poorer OS (OS: 32% vs. 53%, log-rank *p*-value = 0.020) (Figure 1b) [27].

Additionally, these inflammatory reactions can be influenced by multiple factors [5]. In our cohort, the various clinicopathological assessments turned out to be significant concerning patient survival. Variables including tumor length > 10 cm (*p* = 0.016), T4 stage (*p* = 0.015), metastasis (*p* = <0.001), and surgery not performed (*p* = <0.001) were significant in the univariate model. Among these, the surgery not performed (*p* = 0.011) and metastasis group (*p* = <0.001) remained significant in the multivariate model analysis, suggesting that the prognosis is not influenced by surgical intervention as a confounder (Table 3) and that prognosis is heavily influenced by these factors rather than the inflammatory markers themselves. This indicates that while systemic inflammation plays a role in cancer progression, its prognostic value may be secondary to more dominant clinical factors, such as metastasis and surgical intervention. Additionally, the absence of significant associations in the independent multivariate analysis of all markers further suggests that they may be less relevant to the specific South Asian population or influenced by other factors not included in this study. Despite the comprehensive analysis, our study had several limitations, including its single-center design and a follow-up period limited to one year, which may have also not captured long-term outcomes, with the potential for selection bias.

In conclusion, while our study underscores the importance of systemic inflammation in the prognosis of EC, the results for other immune-inflammatory markers were contrasting compared to the available literature. This discrepancy suggests the need for further large-scale studies to confirm these findings. Additionally, exploring the underlying mechanisms linking systemic inflammation to cancer progression and assessing the relevance of these markers in diverse populations could provide deeper insights and enhance the stratification of patients, ultimately guiding personalized treatment strategies for EC.

## Figures and Tables

**Table 1:** Optimal cut-off values of inflammatory markers for overall survival prediction.

Inflammatory Markers	AUC (95% CI)	Cut-Off Values	Sensitivity (%)	Specificity (%)
Neutrophil to lymphocyte ratio (NLR)	0.56 (0.46–0.65)	3.6	54.0	66.1
Platelet to lymphocyte ratio (PLR)	0.52 (0.41–0.60)	198.4	44.8	64.4
Lymphocyte monocyte ratio (LMR)	0.53 (0.43–0.63)	4.3	66.7	47.5
C-reactive protein albumin ratio (CAR)	0.57 (0.47–0.67)	0.17	81.6	33.9
RDW to platelet ratio (RPR)	0.58 (0.48–0.67)	5.2	75.9	42.4
Systemic immune inflammation (SII) index	0.58 (0.48–0.67)	858.0	69.0	52.5
Systemic inflammation score (SIS)	0.57 (0.47–0.66)	2	62.1	52.5
Modified Glasgow prognostic score (mGPS)	0.56 (0.46–0.65)	2	39.1	72.9

AUC = area under the curve; CI = confidence interval.

**Table 2:** Demographic and clinicopathological characteristics of esophageal cancer patients (*n* = 146).

Characteristics	Total *n* (%)	Alive (*n* = 59)	Expired (*n* = 87)	*p*-Value *
**Age in years, Median (Q_1_–Q_3_)**		45 (35–58)	48 (35–60)	0.875
**Gender**				
Male	69 (47.3)	32 (54.2)	37 (42.5)	0.164
Female	77 (52.7)	27 (45.8)	50 (57.5)	
**Histopathology**				
Adenocarcinoma	39 (26.7)	17 (28.8)	22 (25.3)	0.637
Squamous cell carcinoma	107 (73.3)	42 (71.2)	65 (74.7)	
**Tumor site**				
Upper thoracic	13 (8.9)	5 (8.5)	8 (9.2)	0.778
Mid thoracic	54 (37.0)	20 (33.9)	34 (39.1)	
Lower thoracic involving the junction	79 (54.1)	34 (57.6)	45 (51.7)	
**Grade of differentiation**				
Well differentiated	15 (10.3)	8 (13.6)	7 (8.0)	0.155
Moderately differentiated	95 (65.1)	41 (69.5)	54 (62.1)	
Poorly differentiated	36 (24.7)	10 (16.9)	26 (29.9)	
**Tumor length**				
<5 cm	44 (30.1)	21 (35.6)	23 (26.4)	0.031
5–10 cm	78 (53.4)	34 (57.6)	44 (50.6)	
>10 cm	24 (16.4)	4 (6.8)	20 (23.0)	
**cT stage**				
T1–T2	19 (13.0)	13 (22.0)	6 (6.9)	0.021
T3	63 (43.2)	25 (42.4)	38 (43.7)	
T4	64 (43.8)	21 (35.6)	43 (49.4)	
**cN stage**				
N0	13 (8.9)	10 (16.9)	3 (3.4)	<0.001
N1	51 (34.9)	29 (49.2)	22 (25.3)	
N2	44 (30.1)	12 (20.3)	32 (36.8)	
N3	38 (26.0)	8 (13.6)	30 (34.5)	
**M stage**				
M0	103 (70.5)	53 (89.8)	50 (57.5)	<0.001
M1	43 (29.5)	6 (10.2)	37 (42.5)	
**Clinical stage**				
I–II	12 (8.2)	7 (11.9)	5 (5.7)	0.012
III	46 (31.5)	25 (42.4)	21 (24.1)	
IV	88 (60.3)	27 (45.8)	61 (70.1)	
**Surgery**				
Performed	41 (28.1)	25 (42.4)	16 (18.4)	0.002
Not performed	105 (71.9)	34 (57.6)	71 (81.6)	
**Neutrophil to lymphocyte ratio (NLR)**				
<3.6	79 (54.1)	39 (66.1)	40 (46.0)	0.017
≥3.6	67 (45.9)	20 (33.9)	47 (54.0)	
**Platelet to lymphocyte ratio (PLR)**				
<198.4	86 (58.9)	38 (64.4)	48 (55.2)	0.266
≥198.4	60 (41.1)	21 (35.6)	39 (44.8)	
**Lymphocyte monocyte ratio (LMR)**				
<4.3	89 (61.0)	31 (52.5)	58 (66.7)	0.086
≥4.3	57 (39.0)	28 (47.5)	29 (33.3)	
**C-reactive protein albumin ratio (CAR)**				
<0.17	36 (24.7)	20 (33.9)	16 (18.4)	0.033
≥0.17	110 (75.3)	39 (66.1)	71 (81.6)	
**RDW to platelet ratio (RPR)**				
<5.2	46 (31.5)	25 (42.4)	21 (24.1)	0.020
≥5.2	100 (68.5)	34 (57.6)	66 (75.9)	
**Systemic immune inflammation (SII) index**				
<858.0	58 (39.7)	31 (52.5)	27 (31.0)	0.009
≥858.0	88 (60.3)	28 (47.5)	60 (69.0)	
**Systemic inflammation score (SIS)**				
0	6 (4.1)	4 (6.8)	2 (2.3)	0.158
1	58 (39.7)	27 (45.8)	31 (35.6)	
2	82 (56.2)	28 (47.5)	54 (62.1)	
**Modified Glasgow prognostic score (mGPS)**				
0	37 (25.3)	20 (33.9)	17 (19.5)	0.112
1	59 (40.4)	23 (39.0)	36 (41.4)	
2	50 (34.2)	16 (27.1)	34 (39.1)	

* *p*-value was calculated by the Mann–Whitney test and Chi-square test.

**Table 3:** Univariate and multivariate Cox regression analysis for the risk factors associated with mortality among patients with esophageal cancer.

Characteristics	Survival	Univariate		Multivariate	
	(%)	HR (95% CI)	*p* Value	HR (95% CI)	*p* Value
**Age in years**		0.99 (0.98–1.01)	0.905	-	
**Gender**					
Male	46.4	Ref			
Female	35.1	0.93 (0.60–1.44)	0.744	-	
**Histopathology**					
Adenocarcinoma	43.6	Ref		Ref	
Squamous cell carcinoma	39.3	0.67 (0.41–1.09)	0.108	0.74 (0.40–1.35)	0.327
**Grade of differentiation**					
Well differentiated	53.3	Ref		Ref	
Moderately differentiated	43.2	1.50 (0.68–3.31)	0.314	1.22 (0.52–2.82)	0.648
Poorly differentiated	27.8	1.80 (0.77–4.21)	0.175	0.95 (0.37–2.45)	0.920
**Tumor length**					
<5 cm	47.7	Ref		Ref	
5–10 cm	43.6	1.08 (0.64–1.81)	0.757	0.95 (0.54–1.67)	0.859
>10 cm	16.7	2.09 (1.14–3.84)	0.016	1.23 (0.62–2.45)	0.551
**cT stage**					
T1–T2	68.4	Ref		Ref	
T3	39.7	1.91 (0.80–4.54)	0.145	1.76 (0.67–4.56)	0.247
T4	32.8	2.91 (1.23–6.88)	0.015	1.82 (0.66–5.03)	0.248
**cN stage**					
N0	76.9	Ref		Ref	
N1	56.9	1.03 (0.31–3.49)	0.957	0.50 (0.14–1.82)	0.298
N2	27.3	1.86 (0.56–6.14)	0.308	0.52 (0.14–2.01)	0.347
N3	21.1	2.17 (0.65–7.21)	0.204	0.52 (0.14–2.01)	0.346
**M stage**					
M0	51.5	Ref		Ref	
M1	14.0	4.35 (2.69–7.02)	<0.001	3.09 (1.66–5.73)	<0.001
**Clinical stage**					
I–II	58.3	Ref			
III	54.3	0.61 (0.23–1.65)	0.335	-	
IV	30.7	1.16 (0.46–2.91)	0.749		
**Surgery**					
Performed	61.0	Ref		Ref	
Not performed	32.4	2.86 (1.65–4.95)	<0.001	2.27 (1.21–4.26)	0.011
**Neutrophil to lymphocyte ratio (NLR)**					
<3.6	49.4	Ref			
≥3.6	29.9	1.09 (0.71–1.68)	0.686	-	
**Platelet to lymphocyte ratio (PLR)**					
<198.4	44.2	Ref			
≥198.4	35.0	1.01 (0.65–1.56)	0.971	-	
**Lymphocyte monocyte ratio (LMR)**					
≥4.3	34.8	Ref			
<4.3	49.1	1.13 (0.72–1.78)	0.600	-	
**C-reactive protein albumin ratio (CAR)**					
<0.17	55.6	Ref		Ref	
≥0.17	35.5	1.62 (0.94–2.79)	0.084	1.14 (0.60–2.17)	0.682
**RDW to platelet ratio (RPR)**					
<5.2	54.3	Ref		Ref	
≥5.2	34.0	1.55 (0.94–2.55)	0.080	1.02 (0.57–1.83)	0.947
**Systemic immune inflammation (SII) index**					
<858.0	53.4	Ref		Ref	
≥858.0	31.8	1.71 (1.08–2.71)	0.022	1.41 (0.78–2.54)	0.257
**Systemic inflammation score (SIS)**					
0	66.7	Ref			
1	46.6	1.55 (0.37–6.53)	0.545	-	
2	34.1	2.07 (0.50–8.55)	0.312		
**Modified Glasgow prognostic score (mGPS)**					
0	54.1	Ref			
1	39.0	1.34 (0.74–2.39)	0.326	-	
2	32.0	1.25 (0.69–2.26)	0.448		

Multivariate model was adjusted for those variables with a *p*-value > 0.25 in the univariate model. HR = hazard ratio; CI = confidence interval.

## Data Availability

The data presented in this study are available on request from the corresponding author. The data are not publicly available due to ethical restrictions.
